# The League of Mercy

**Published:** 1911-12-23

**Authors:** 


### THE LEAGUE OF MERCY

### ONE HUNDRED AND EIGHTY THOUSAND POUNDS FOR THE HOSPITALS.

The twelfth annual meeting of Presidents of the League of Mercy was held at St. James's Palace on Friday, December 15, 1911, under the presidency of the Lord Farquhar, G.C.V.O., and he was supported on the platform by H.R.H. the Duchess of Albany, who made a numerous presentation of the Order of Mercy. There was a large attendance.

The following accepted the invitations to be present :—H.H. Princess Victoria of Schleswig-Holstein, Dora Countess of Chesterfield, the Countess of Clarendon, the Viscountess Gage, the Viscountess Midleton, the Lady De L'Isle and Dudley, Gertrude the Lady Decies, the Lady Farquhar, the Dowager Lady Lamington, the Lady Willingdon, the Hon. Mrs. G. Lawson Johnston, the Hon. Mrs. Leveson-Gower, the Hon. Emily St. John, the Hon. Mrs. Hanbury Lennox, the Hon. Mrs. Wood of Hengrave, Lady Alexander, Lady Barker, Lady Bucknill, Lady Burdett, Lady Clayton, Lady Collins, Lady Dimsdale, Lady Edridge, Lady Faudel Phillips, Lady Somerset, the Earl Cadogan, K.G.; the Earl of Clarendon, G.C.V.O.; the Viscount Hill, the Viscount Gage, Col. the Rt. Hon. Mark Lockwood, C.V.O., M.P.; Sir Fitzroy D. Maclean, Bart., K.C.B.; the Lord Lamington, G.C.M.G., G.C.I.E.; the Lord Cowdray of Midhurst, the Rt. Hon. W. Ellison-Macartney, the Rt. Hon. James Round, Sir Kenneth Matheson, Bart.; Sir George Alexander, Lieut.-Col. Sir Adelbert C. Talbot, K.C.I.E.; Sir M. M. Bhownaggree, K.C.I.E.; Sir William J. Bull, M.P.; Sir Walter Johnson, Sir Edward D. Stern, Alderman Sir Charles Wakefield, Rear-Admiral Leicester Keppel, Col. Colvin, C.B.; Capt. G. R. B. Drummond, M.V.O.; Col. Henry G. Thompson, M.D.; Major W. A. Adam, Capt. Speer, the Rev. W. H. Hornby Steer, the Rev. A. Welsh Owen, Mrs. H. Beecroft, Miss Flora Bischoff, Miss M. S. Carter, Mrs. M. Hildred L. Causton, Miss Maud Chaplin, Mrs. Mateo Clark, Mrs. H. H. Clutton, Mrs. Denny Cooke, Mrs. E. M. Corner, Mrs. Edmunds, Mrs. L. de Rothschild, Mrs. W. Ellison-Macartney, Miss Helen M. Enthoven, Mrs. Tyrrell Giles, Miss Tyrrell Giles, Mrs. W. R. Graham, Mrs. Greer, Mrs. Hamilton, Mrs. John Hamilton-Evans, Miss Harrington, Mrs. R. L. Harrison, Mrs. Carl Hentschel, Miss Hickley, Mrs. Hodson, Mrs. E. Murray Ind, Miss Constance G. Johnson, Mrs. Leicester Keppel, Mrs. Lefroy, Mrs. Lewis, Mrs. Lockwood, Miss M. J. Lovell, Miss Lowe, Mrs. Arthur Lyell, Mrs. McIntosh, Miss Maude E. Meadows, Mrs. Metzler, Mrs. Freeman Murray, Mrs. S. Elland Norton, Mrs. Pendred, Mrs. Pigeon, Mrs. Price, Miss Adeline Raffles, Mrs. Rodwell, Miss Rodwell, Mrs. T. J. Russell, Miss Ada St. Quintin, Miss Hilda M. Seebohm, Mrs. Jonathan Smith, Miss Stevens, Miss Cecilia E. Stone, Mrs. Sidney Straus, Miss Theobald, Mrs. Edgar Tyzack, Mr. W. A. Brown, Mr. W. Stanford Gates, Mr. C. Tyrrell Giles, K.C.; Mr. James Godding, Mr. Frederick Green, Mr. A. H. Greening, Mr. Carl Hentschel, Mr. Alderman George Hinds, Mr. Sidney Hoffnung-Goldsmid, Mr. E. Murray Ind, Mr. H. W. Keay, Mr. W. H. Cortlandt Mahon, Mr. J. A. Paton, Mr. Thomas Phillips Price, Mr. John Sadler, Mr. J. F. Sarjeant, Mr. Alexander T. Scott, Mr. Montagu Sharpe, Mr. B. S. Straus, Mr. Philip F. Walker, Mr. F. A. White. There were also present : Lord Wolverton, Sir Henry Burdett, K.C.B., K.C.V.O.; Sir J. Harrison, M.V.O.; Mr. E. W. Wallington, C.V.O., C.M.G.; Mr. Perceval A. Nairne, and Colonel H. B. Hamilton.

### Message from the King.

Mr. E. W. Wallington, C.V.O., C.M.G., Honorary Registrar of the Order, communicated to the meeting of Presidents the following message from his Majesty :—

"India, December 14, 1911.

"Please convey to the League of Mercy meeting to-morrow the King's sincere congratulations on successful year.

"His Majesty feels sure that the workers will continue their generous and untiring efforts.

"His interest in, and sympathy with, the League is as great as ever.

(Signed) "Stamfordham."

Lord Farquhar said he felt sure all the Lady Presidents and Presidents would give as much assistance as hitherto, loyally serving the Lady Grand President, Princess Alexander of Teck. He (the Chairman) regretted to say that the Grand President and the Lady Grand President were at present abroad, but they had most kindly entrusted the presentation of the Order of Mercy to the hands of H.R.H. the Duchess of Albany, herself a holder of the Order, and an ardent supporter of the League since its formation. At present H.R.H. had charge of three districts of the League. He took the opportunity of thanking the Princess Victoria of Schleswig-Holstein for the work which, for so many years, she had accomplished, as shown by the great success she had had in her districts—Oxford, Buckingham, Berkshire, Hampshire. All present would remember the concert which was given in the Albert Hall, and many of the Presidents and Vice-presidents had proposed the holding of a similar kind of entertainment this year; but the conclusion arrived at was that, owing to the Coronation and the consequent festivities, it would be wiser not to. A proposition was now put forth to have such an event next year, and he thought this opportunity was a favourable one for ascertaining views on the desirability of it. He suggested that those who held definite views on the matter should write to the Secretary at the offices of the League, and indicate what assistance they would be prepared to give to it. It only remained for him to thank all the Presidents for the good work they had done, and to wish them continued success in the future. The amount collected by the League this year, as far as could be ascertained, was £19,306. (Applause.) Last year the amount collected was £21,186, a decrease of £1,880. The grants made to country hospitals this year amounted to £2,300. Last year the corresponding sum was £2,100. He felt compelled to state—though unwillingly—that the result was not quite as good as the League would have liked. All sorts of reasons had been given for the reduced amounts, and Colonel Kempster had received two basketsful of letters of that kind. (Laughter.) He would not enter into the reasons, as some of them were of a political character. Surely, the name "The League of Mercy" ought to appeal to all; it was a beautiful name, and one which would continue to exercise a good influence over the hearts of those who were really interested in the poor. (Applause.)

### The Business of the Year.

Viscount Hill moved, "That the Right Honourable the Lord Farquhar, G.C.V.O., be appointed Chairman, Mr. Frederick Green, J.P. Deputy-chairman, and Colonel H. B. Hamilton Hon. Secretary respectively." In doing so he said it was only recently that he had the honour of being appointed President of one of the branches of that great League, and so it would be hopeless for him to attempt to inform the meeting of the fine work which had already been done. He did not hesitate to say that a still greater work awaited the League in the future, as there would be more difficulties to contend with. Thus it was increasingly necessary to have at the head of the League men of enthusiasm, who would put of their best into the work, and give the Presidents a lead in all parts of the country.

Mr. Philip Walker seconded, remarking that the gentlemen whose names had been read out were a very great asset to the League. He was sure that with those gentlemen in office the organisation would make even greater strides in the future than it had in the past.

The resolution was carried unanimously.

The Right. Hon. W. Ellison-Macartney proposed, "That seven representative members of the Executive Committee be appointed: The Right Hon. The Lord Farquhar, G.C.V.O., Colonel H. B. Hamilton, Mr. Frederick Green, J.P., Mrs. R. L. Harrison, Mr. Edward Murray Ind, D.L., J.P., Lady Dimsdale, and Mr. Montagu Sharpe, D.L." He said he need only add he was confident everyone present would agree that the interests of the League could safely be entrusted to those whose names he had proposed.

Lieutenant-Colonel Sir Adelbert Talbot, K.C.I.E., seconded, remarking that the League was very fortunate in having secured the services of the seven representative members whose names were read out, and so long as they were willing to offer their services the League could not do better than prolong their successful tenure of office.

Carried unanimously.

### Presentation of the Order of Mercy by H.R.H. the Duchess of Albany.

H.R.H. the Duchess of Albany then made about one hundred presentations of this distinguished Order to officers and zealous workers who are members of the League of Mercy.

### The Treasurer's Statement.

Sir Henry Burdett, K.C.B., K.C.V.O., Hon. Treasurer, said the year had been a good one for the League, but not for the Treasurer. Those connected with the League had had to work harder; they had had to face more difficulty, more annoyance, more diversions, more abstractions than in any of the preceding eleven years of the League's activity. But still, he said, the year had been good, because the Presidents could not otherwise know what their organisation was worth; its weight and force could not be adequately appreciated until it came upon troublous times. This year had been a difficult and bad one, but yet the work had been done so well that, whereas in eleven years £180,000 had been distributed among the hospitals, or about £18,000 a year, this year £19,200 had been sent, although only £19,385 had been collected. As Treasurer, he always took upon himself the responsibility of keeping some money in hand against a rainy day, so that the gifts could be equalised, and the contributions to the hospitals made reliable and steady. To those who questioned the wisdom of keeping a little money in hand he hoped the practical answer he was able to give to-day would prove satisfactory. (Applause.) In the counties where there had been a greater strain owing to Lord-Lieutenants and others raising special funds, the League had done exceedingly well. The four counties under H.H. Princess Victoria's control produced this year most satisfactory results; indeed, the Princess was one of the most zealous, if not the most zealous, of workers. He was also most gratified to record that although ninety-two Vice-Presidents had resigned, the Lady Presidents, Presidents, and their co-workers had been able to recruit 139 new people to take office, a gain of forty-seven Vice-Presidents for the year. (Applause.) That was very remarkable in a year like this. Therefore, because the work next year was going to be hard, and more necessary than it had ever yet been, for reasons which he would explain later, he hoped those present would go forth from that meeting full of courage, thankfulness, and hope.

With his assurance that, based upon experience of forty years, he did congratulate the Lady Presidents and Presidents, and thank them for the splendid results they had achieved. Sir Henry then read through the list of amounts collected in the various districts, saying an encouraging word here and there, or halting to pay a special tribute to work in exceptional difficulties. The complete list of the sums raised by each district will be published at the end of the financial year. He said he had the privilege during the year of being present at a drawing-room meeting where Sir Beerbohm and Lady Tree were present. Lady Tree opened the League's report and gave a most stirring address, with the result that a larger sum was raised than at any other drawing-room meeting. How did she do it? The answer would be found by reading the first chapter of St. Matthew's Gospel. She took the report, mainly a book of names, as an index of progress in what he was told yesterday by one of the hardest workers among the poor in the Metropolis was one of the most useful and valuable organisations in this country. It might encourage all the Presidents to hear that the average contribution this year from the League Divisions was some £214. (Applause.) The function of the League was to spread knowledge concerning the work of hospitals, and of the way in which that work was done. It was their care to take charge of the sick workers, and all present were aware, of their own knowledge, how great had been the service rendered by the League of Mercy by means of the £180,000 contributed.

### At the Parting of the Ways.

Such co-operation had never been more needed than at the present day, as we were now at the parting of the ways in hospital matters, and it was the truth that the artizans and workers might lose hospital care. Owing to the constant changes which had recently occurred, it was very difficult for even a student to know where we now stand in legislative matters. But all who worked for hospitals would agree, whatever their political opinions, that a crisis was now imminent, and this must tend to diminish the number of free hospital beds, which had heretofore been the salvation and the safety of the working classes all over the country. How much this was so would be realised from the fact that, owing to the improvements in treatment carried out in modern hospitals, the stay of patients requiring surgical aid and the need for operations had been reduced from thirty-five days to about eighteen days, as was proved by the figures. What did that mean? That a million working days in London alone had been added per annum to the earning capacity of its working-men. (Applause.) It must be realised by all who went about engaged in this work that they had before them a most difficult task, and that the legislation now to be faced may result in a large diminution of hospital bed accommodation. Fairy tales were not facts, and the difficulties ahead were momentous.

### The Artizans and Workmen in Danger.

He had just received a telegram from the Chairman of the Cardiff Hospital, in which he said the member for Merthyr stated that several direct contributions would be made to hospitals from approved societies and local Health Committees. Last night, in the House of Lords, Lord Haldane replied to Lord Byron, when he raised the question of institutional benefit which would have given under the Bill, if Lord Byron's amendment had been accepted, hospital beds for the protection of the working-men. The reply of Lord Haldane showed that he did not understand the Act. And he (Sir Henry) could say in reference to the remark of the member for Merthyr, that it was not the case, as Mr. Jones supposed, that there were direct contributions which would be made to hospitals by approved societies or local Health Committees in the sense that they would be material. They could not be so. He (Sir Henry) was not there to demonstrate that on the present occasion, but he was present as one who had devoted most of the leisure of his life to voluntary hospitals, and who believed that the most sacred duty a man or a woman could have was to do his utmost to take care that those who were the bread-winners of this country should have adequate hospital treatment when they needed it, so that those dependent upon them might have the best opportunity of being cared for in this England of ours to-day. Disaster was threatening, and the sooner that was clearly understood the better, because if it were understood quickly the case was so strong that he made bold to believe that, whatever legislation might be attempted in the next Session of Parliament, there would certainly be an amending Act, which would provide ample safeguards by making provision for the breadwinner against the day of his sickness, so that, when he needed it, he should be guaranteed hospital care. He apologised for having said so much, but the crisis was acute. It had been his aim to gain a clear understanding of the Bill, and he would not have thought he had discharged his duty to his fellow-countrymen if he had not earnestly raised his voice to try to get everybody, before they said there was adequate hospital provision under the Bill, to at least study and try to understand the Act. If he had not understood it himself, he hoped an authoritative official statement would be forthcoming, showing in what way and how adequate hospital accommodation could be provided. He congratulated the Presidents most heartily on their work in a difficult year, the difficulties having made it the best year the League had experienced. It established the League as a permanent institution of great value which no calamity would shake. This year all workers would be encouraged by the knowledge that whatever services they had rendered in the past by personal effort, those services would be enhanced in the future owing to the increased necessities of those whose interests the League represented and tried to safeguard. (Applause.)

### The Work in 1911 and 1912.

Sir J. Harrison, M.V.O., said it usually fell to the lot of his colleague, Sir William Collins, to place before the meeting certain details of the work of the League during the year, but public duties prevented his coming; this was the only occasion on which he had not been present, and he asked him (the speaker) to make his apologies for absence to H.R.H. the Duchess of Albany. A fortnight still remained of the present year, and he believed that in this period some of the deficit which had been alluded to would have been made up. During the year there had been forty receptions of which the Central Office had knowledge, but he believed there had been many others, of which notice had not been received. One was specially deserving of mention—namely, that given by Lord and Lady Farquhar, who entertained about a thousand workers in their beautiful house and grounds at White Lodge. It was an honour to them, but it was a greater honour to all those who were present, that their Majesties the King and Queen, amid their many engagements in this busy year, graced the reception by their presence. (Applause.) There were also two large gatherings which H.R.H. the Duchess of Albany held at Claremont, and a large and very important meeting at the Mansion House, arranged by Lady Dimsdale, at which the Lord Mayor took the chair, and Sir Frederick Treves spoke. Recently there was a meeting at Folkestone, at which Sir Squire Bancroft presided and spoke, and at East Marylebone the Hon. Mrs. Lawson Johnson gave a meeting in her house, at which Sir Herbert Beerbohm Tree, Lady Tree, and Sir James Crichton Browne spoke. At Mid-Essex Mr. and Mrs. Murray Ind had a large meeting at their house; at South Paddington Mrs. Edwin Mackintosh had a meeting at 79 Lancaster Gate, at which Sir Dyce Duckworth spoke. In addition, there were many entertainments. The League must feel very grateful to all those ladies and gentlemen who had so kindly held meetings and entertainments. It was obvious that in a large organisation like the League changes must come; and owing to deaths—which all deplored—and to other causes it had been necessary in the past year to appoint several new Lady Presidents and Presidents. There still remained districts for which a Lady President or President was required; and if any President could help the Secretary to select workers who would really take an interest in the matter in those districts, the League would be most grateful. The fact that the Secretary had received two basketsful of letters giving reasons for lessened collections might be looked upon as a sign of depression, but he thought there was no need for depression. As the Hon. Treasurer had mentioned, next year would be more difficult for the League. He did not wish to hurt anybody's feelings—and it would not apply to anybody present—but he could understand the discouragement felt when people simply refused to subscribe to the League and help their fellows in this way: there was no more to be said to them; they were the Levites who passed by on the other side. There were other people, who accepted the work—perhaps they were appointed early in the year—and when they were asked in October what was the result of their efforts they said they were sorry, but they had forgotten about it, and would work well next year. That was distinctly discouraging. He believed the difficulties for next year had been somewhat exaggerated. But if the difficulties proved greater, efforts must be redoubled. The need for the future would be as great as it had been in the past. The reasons for which King Edward founded the League in 1899, when he said he desired the interest of a large number of new persons in the work of hospitals, and subscriptions from among those who did not subscribe at all, were now as great as ever. Those present would agree that they were bound in honour, and by the love they bore their great King, to carry on the work which he set them to do. It would also be agreed that they could not let the King return from his Imperial mission in India, especially after the gracious message he had sent over to the League, and find that there was any need for discouragement or falling-off in results. He felt sure that as time went on difficulties would be swept away, and it would be seen how easy it was to encounter them if it were set about in the right way. Sometimes it was said to those at the head office, "We cannot go to the working-man." All he could reply was that only yesterday he had in his hand details of certain districts; and in one district upwards of £100, and in another £60, had been collected in penny weekly contributions.

### Votes of Thanks.

Sir Charles Wakefield proposed, "That the Chairman be thanked for his kindness in taking the chair, and Mr. Griffiths for his address." He said he was sure the vote would commend itself to the judgment of those present. Lord Farquhar was ubiquitous in the field of charity, and his ear was ever attentive to the cry of the widow and the orphan. A deep debt of gratitude was also owing to Mr. Griffiths for his magnificent address. Mr. Griffiths had gripped the hearts and imaginations of his hearers, and the address would be a stimulus to the Presidents in their future work for the League.

Alderman George Hinds, in seconding the resolution, said he had not previously envied America one of her citizens, but he had that feeling about the Hon. John Griffiths. He wished Mr. Griffiths could belong to the League of Mercy and attend the meetings, for then the subscriptions would probably be doubled.

The resolution was carried with much cordiality.

The Chairman, in acknowledging the vote, said he felt sure that when King George returned to this country and saw the result of the year's working he would say, as Sir Henry Burdett had already said, that this year had been not the worst but probably the best they had had, considering the circumstances with which the workers had had to deal. It was gratifying that the number of Vice-Presidents and Lady Vice-Presidents had been increased during the year; more had been appointed than had resigned. He thanked Mr. Griffiths for his most eloquent speech; he wished he had anything like the same eloquence. He alluded gratefully to the excellent work which had been done by Colonel Kempster. Nothing could exceed his attention, and those who went to see him could not receive greater courtesy, although he had to see a large number of people. Nothing could be more excellent or praiseworthy than the whole of his management.

